# Development and validation of the Topical Steroid and Moisturizer Adherence Scale

**DOI:** 10.1016/j.jdin.2026.01.005

**Published:** 2026-01-30

**Authors:** Cheung Ying Shan, Du Ruochen, Tan Ken Wei, Valencia Long, Qasrina Lim, Nisha Suyien Chandran, Chris Tan, Jose M. Valderas, Phillip Phan, Ellie Choi

**Affiliations:** a Division of Dermatology, Department of Medicine, National University Hospital, Singapore; b Biostatistics Unit, Yong Loo Lin School of Medicine, National University of Singapore, Singapore; c Saw Swee Hock School of Public Health, National University of Singapore, Singapore; d Department of Medicine, Yong Loo Lin School of Medicine, National University of Singapore, Singapore; e Department of Family Medicine, National University Healthcare System, Singapore; f Centre for Research in Health System Performance, Yong Loo Lin School of Medicine, National University of Singapore, Singapore; g Carey Business School, Johns Hopkins University, Baltimore; h Division of Geriatric Medicine and Gerontology, Department of Medicine, Johns Hopkins University, Baltimore; i Mind Science Centre, Yong Loo Lin School of Medicine, National University of Singapore, Singapore

**Keywords:** adherence, behavioral, eczema, health outcomes, health services research, moisturizers, Patient reported measures, patient-centered care, psoriasis, quality of life, questionnaire validation, topical steroids

*To the Editor:* While dermatologic outcomes are often compromised by nonadherence to prescribed topical therapies,^1^ the ability to measure and track topical nonadherence is limited by the lack of validated instruments. Existing measures such as tube weighing^2^ and smart-cap trackers^3^ are costly and limited by variability in treatment requirements that vary with disease severity. We developed and prospectively validated a 7-item tool—Topical Steroid and Moisturizer Adherence Scale (TSMAS)—a simple and practical patient-reported measure to assess adherence to topical corticosteroids (TCS) and moisturizers.

Items were developed based on literature search of 22 articles and expert opinion, comprising questions on TCS adherence (frequency, quantity, potency, and overall use) and moisturizer adherence (frequency, quantity, and overall use). Items employed Likert-type response options with a 30-day recall period.

Face validity and cognitive debriefing were performed in 5 patients, followed by validation in 119 dermatology outpatients from the National University Hospital, Singapore, between August 2022 and May 2024. Patients aged 16 to 94 years (median 42 years), prescribed TCS and moisturizers for a chronic inflammatory skin disease completed the questionnaire at baseline and 1 month (Table I). To support construct validity, the questionnaire included patient-reported outcomes and clinician assessments (Table II). Standard psychometric methods were used to assess internal consistency, test–retest reliability, construct validity, and responsiveness.

**Table I:** Baseline characteristics of participants (*N* = 119)

Characteristic	Value
Age, mean (SD)	44.5 (19.2) y
Sex, *n* (%)	
Male	62 (52.1%)
Female	57 (47.9%)
Ethnicity, *n* (%)	
Chinese	82 (68.9%)
Malay	18 (15.1%)
Indian	9 (7.6%)
Other	10 (8.4%)
Highest education level, *n* (%)	
Primary school	2 (1.7%)
Secondary school	22 (18.5%)
Junior college/ITE	43 (36.1%)
Bachelor’s degree	41 (34.5%)
Master’s/Doctorate	11 (9.2%)
Disease condition, *n* (%)	
Eczema∗	92 (77.3%)
Prurigo nodularis	1 (0.8%)
Autoimmune blistering disease	4 (3.4%)
Psoriasis	20 (16.8%)
Others (Jessner’s lymphocytic infiltrate, Cutaneous lupus)	2 (1.7%)

∗ Includes all eczema variants (Atopic dermatitis, hand and feet eczema, discoid eczema, asteatotic eczema).

**Table II:** Overview of questionnaire items

Constructs	Questions	Answer options
Topical Steroid and Moisturizer Adherence Scale (TSMAS)		
Adherence to Topical Steroids (TCS)	Compared to what I’ve been prescribed and asked to use, I am	Always adherent; Sometimes adherent; Seldom adherent; Nonadherent/I don’t use
	Compared to how often I am supposed to apply the steroid creams prescribed to me, I use it	Much more frequently; Slightly more frequently; As prescribed; Slightly less frequently; Much less frequently; I don’t use
	Compared to how much I am supposed to apply the steroid creams prescribed to me, I use a	Much larger amount; Slightly larger amount; As prescribed; Slightly smaller amount; Much smaller amount; I don’t use
	Compared to what I was prescribed, most of the time, I use	More of the stronger steroid cream; As prescribed; More of the weaker steroid cream; I don’t use; Not relevant (single strength)
Adherence to Moisturizers	Compared to what I’ve been prescribed and asked to use, I am	Always adherent; Sometimes adherent; Seldom adherent; Nonadherent/I don’t use
	How frequently do you use moisturizers?	Twice daily or more; Once daily; 2-4 × weekly; Weekly or less; I don’t use
	How much moisturizers do you apply?	Large and thick amount; Moderate amount; Small and thin amount; I don’t use

Baseline patient reported tools for evaluation of psychometric properties		
QoL impairment (Skindex mini)		
Anxiety (GAD-7)		
Social desirability		
Self-reported severity		
Self-reported body surface area (BSA)		
Desire for improvement		
Steroid adherence barriers		
Moisturizer adherence barriers		
Constructs assessed at 1 mo follow up		
Adherence measure—steroids and moisturizers		
Self-reported severity		
Self-reported body surface area (BSA)		
Information provided by the physician at baseline		
BSA		
Investigator’s global assessment (IGA)		
Patient’s knowledge of steroid cream (name and potency)		
Patient’s technique of application		
Doctor’s estimated quantity of TCS usage (fraction of used vs should have used)		

Item-level ceiling effects were observed, with >50% indicating full adherence for questions on prescribed steroid quantity and potency, overall moisturizer adherence and frequency of application. However, the domain-level summed scores did not exhibit ceiling effects. Internal consistency was good for TCS (α = 0.86) and modest for moisturizer-related items (α = 0.66). Confirmatory factor analysis (CFA) showed acceptable fit for a 2-factor model with a Comparative Fit Index (CFI) of 0.93, Tucker-Lewis Index (TLI) of 0.89 and Standardized Root Mean Square Residual (SRMR) of 0.078, though the Root Mean Square Error of Approximation (RMSEA) of 0.116 was suboptimal suggesting room for further optimization (detailed results in Supplementary File, available via Mendeley at https://data.mendeley.com/datasets/ygvc7zmj85/1).

Convergent validity was demonstrated with physician-rated adherence (ρ = 0.34), perceived benefits (ρ = 0.26), and desire for skin improvement (ρ = 0.38). In contrast, weak or non-significant correlations with anxiety (ρ = 0.18), quality of life (ρ = 0.02) and education (ρ = −0.04) supported discriminant validity. There was no correlation between adherence and social desirability, suggesting no interviewer or social desirability bias.^4^

Test–retest reliability was moderate for the TCS domain (intraclass correlation coefficient: 0.762) and for the moisturizer domain (0.569). Responsiveness was moderate, with standardized mean response (SMR) of 0.585 and −0.596 for improved and reduced adherence in the steroid domain, and 0.360 and −0.385, respectively, in the moisturizer domain.

The TSMAS demonstrates acceptable reliability and validity for measuring adherence. It offers a simple, low-cost way to detect nonadherence and guide management. Study limitations include a non-normal item distribution, a small item pool and the absence of a gold standard criterion measure precluding criterion validity evaluation. A ceiling effect also limited discrimination at higher adherence levels. Future work can explore refinements to response options (see proposed questionnaire in Supplementary File, available via Mendeley at https://data.mendeley.com/datasets/ygvc7zmj85/1), optimization through item refinement, model re-specification, and evaluation across broader populations to establish clinically meaningful thresholds.^5^

## Conflicts of interest

None disclosed.
